# RETRACTION: Effectiveness of Chlorhexidine Versus Povidone‐Iodine for Preventing Surgical Site Wound Infection: A Meta‐Analysis

**DOI:** 10.1111/iwj.70318

**Published:** 2025-03-06

## Abstract

**RETRACTION:** WangP. , WangD. , and ZhangL. , “Effectiveness of Chlorhexidine Versus Povidone‐Iodine for Preventing Surgical Site Wound Infection: A Meta‐Analysis,” International Wound Journal 21, no. 2 (2024): e14394, 10.1111/iwj.14394.PMC1082812237752735

The above article, published online on 26 September 2023, in Wiley Online Library (http://onlinelibrary.wiley.com/), has been retracted by agreement between the journal Editor in Chief, Professor Keith Harding; and John Wiley & Sons Ltd. Following an investigation by the publisher, all parties have concluded that this article was accepted solely on the basis of a compromised peer review process. The editors have therefore decided to retract the article. The authors did not respond to our notice regarding the retraction.

**RETRACTION:** P. Wang , D. Wang , and L. Zhang , “Effectiveness of Chlorhexidine Versus Povidone‐Iodine for Preventing Surgical Site Wound Infection: A Meta‐Analysis,” International Wound Journal 21, no. 2 (2024): e14394, 10.1111/iwj.14394.PMC1082812237752735

The above article, published online on 26 September 2023, in Wiley Online Library (http://onlinelibrary.wiley.com/), has been retracted by agreement between the journal Editor in Chief, Professor Keith Harding; and John Wiley & Sons Ltd. Following an investigation by the publisher, all parties have concluded that this article was accepted solely on the basis of a compromised peer review process. The editors have therefore decided to retract the article. The authors did not respond to our notice regarding the retraction.

